# Evaluation of screening practices for primary hyperaldosteronism by specialists and general practitioners: an observational, cross-sectional study

**DOI:** 10.20945/2359-4292-2023-0211

**Published:** 2024-03-21

**Authors:** Giselle Fernandes Taboada, Aline Barbosa Moraes, Leonardo Vieira

**Affiliations:** 1 Universidade Federal Fluminense Departamento de Clínica Médica Hospital Universitário Antônio Niterói RJ Brasil Universidade Federal Fluminense, Departamento de Clínica Médica, Hospital Universitário Antônio Pedro, Niterói, RJ, Brasil; 2 Universidade Estácio de Sá/IDOMED Disciplina de Clínica Médica Rio de Janeiro RJ Brasil Disciplina de Clínica Médica, Universidade Estácio de Sá/IDOMED, Rio de Janeiro, RJ, Brasil; 3 Universidade Federal do Rio de Janeiro Departamento de Clínica Médica Hospital Universitário Clementino Fraga Filho Rio de Janeiro RJ Brasil Universidade Federal do Rio de Janeiro, Departamento de Clínica Médica, Hospital Universitário Clementino Fraga Filho, Rio de Janeiro, RJ, Brasil

**Keywords:** Hyperaldosteronism, primary hyperaldosteronism, hypertension, screening, diagnosis

## Abstract

**Objective:**

Despite its recognized importance, primary hyperaldosteronism (PHA) remains an underdiagnosed condition in clinical practice. The objective of the present study was to evaluate PHA screening practices by general practitioners and specialists in endocrinology and cardiology. Subjects and methods: This cross-sectional, observational study invited physicians to respond voluntarily to an online survey. The survey collected the respondents’ sociodemographic data and answers to five hypothetical clinical cases meeting Endocrine Society criteria for PHA screening.

**Results:**

In all, 126 physicians responded to the online survey. Endocrinologists were the specialists who most often chose PHA screening, although the screening rates were overall low, ranging from 36.5% to 92.9%, depending on the case and the respondents’ specialty. The survey also assessed the reasons for not choosing PHA screening, which included limited availability of tests within the public health services, interference of antihypertensive medications on hormone levels, and failure to identify the screening indication. Being an endocrinologist was an independent predictor for choosing PHA screening for the patients in Cases #1 and #5 (p = 0.001 and p = 0.002, respectively).

**Conclusion:**

Endocrinologists were the specialists who most often chose PHA screening, although the screening rates were overall low among all specialists. These findings highlight a need for continuing medical education programs addressing PHA screening and making the diagnosis of PHA more present in the daily clinical practice of physicians treating patients with hypertension.

## INTRODUCTION

Primary hyperaldosteronism (PHA) carries a higher risk of cardiovascular and renal outcomes than essential hypertension, even when patients are matched for blood pressure levels, age, sex, and comorbidities (1,2). The excess cardiovascular risk in patients with PHA is only reduced when renin levels "escape" suppression by aldosterone through treatment with mineralocorticoid antagonists at appropriate doses or adrenalectomy (3). This emphasizes the importance of identifying the presence of PHA for the implementation of proper therapy beyond the control of blood pressure levels.

Despite its recognized importance, PHA remains an underdiagnosed condition in clinical practice. Studies show that attending physicians fail to carry out screening tests even in situations in which PHA is highly prevalent, like resistant hypertension and spontaneous or diuretic-induced hypokalemia (4-8). This may be due to a lack of awareness of PHA prevalence and the importance of screening and diagnosing this condition for the implementation of adequate treatment, although other factors may also be involved.

Although the initial screening for PHA seems simple (*i.e.*, calculation of the aldosterone-to-renin ratio), it becomes complicated when considering the numerous factors that may interfere with the results (9). Indeed, the interpretation of the results can be confusing during treatment with antihypertensive drugs affecting the renin-angiotensin-aldosterone system (RAAS). Many clinicians are intimidated by this challenge and by the laborious prospect of replacing antihypertensive drugs that are commonly used with others with less effect on RAAS in order to screen for PHA (10).

The objective of the present study was to evaluate the PHA screening practices by general practitioners and specialists in endocrinology and cardiology. Understanding these aspects of PHA screening may help design continuing medical education programs contributing to increasing PHA awareness.

## PARTICIPANTS AND METHODS

This was a cross-sectional, observational study with data obtained from an online survey. Informed consent was obtained from all participants. The study was approved by the local Ethics Committee in July 2021 (CAAE 46378921.6.000.5243).

### Participants

Physicians members of the Brazilian Society of Internal Medicine of Rio de Janeiro (*Sociedade Brasileira de Clínica Médica – RJ*), Brazilian Society of Endocrinology and Metabolism of Rio de Janeiro (*Sociedade Brasileira de Endocrinologia e Metabologia – RJ*), and Rio de Janeiro State Society of Cardiology (*Sociedade de Cardiologia do Estado do Rio de Janeiro*) were invited to participate in the study. Secretaries from each society emailed the invitation to lists of members, along with a link to respond anonymously to the survey. The study had no exclusion criteria.

The sample size was calculated using confidence intervals for population proportions based on an estimated population of 2,200 general practitioners, 2,000 cardiologists, and 800 endocrinologists in the state of Rio de Janeiro, according to information provided by each society. With a margin of error of 5% and a confidence level of 95%, the estimated sample size was 99 respondents.

## METHODS

The online survey was created on the Google platform. The survey collected the respondents’ sociodemographic data (including age, time since graduation and specialization, specialty, and whether they worked in the public and/or private sectors) and presented five hypothetical clinical cases. The five cases, described below, met Endocrine Society criteria for PHA screening (9) (Table 1):

- Case #1: The patient is a 48-year-old woman with hypertension using hydrochlorothiazide 25 mg/day and losartan 50 mg/day. On routine laboratory tests, her potassium is 3.4 mmol/L (3.5-5.5 mmol/L). On physical examination, her blood pressure is 114/78 mmHg (right arm, lying down and sitting up).
- Case #2: The patient is a 56-year-old man with hypertension using losartan 100 mg/day, hydrochlorothiazide 25 mg/day, amlodipine 10 mg/day, and atenolol 50 mg/day. On physical examination, his blood pressure is 130/84 mmHg (right arm, lying down and sitting up).
- Case #3: The patient is a 47-year-old woman with hypertension using losartan 100 mg/day, hydrochlorothiazide 25 mg/day, amlodipine 20 mg/day, and atenolol 100 mg/day. On routine laboratory tests, her potassium is 3.1 mmol/L (3.5-5.5 mmol/L). On physical examination, her blood pressure is 164/112 mmHg (right arm, lying down and sitting up).
- Case #4: The patient is a 52-year-old woman with hypertension using losartan 100 mg/day, hydrochlorothiazide 25 mg/day, and amlodipine 10 mg/day. On physical examination, her blood pressure is 144/92 mmHg (right arm, lying down and sitting up).
- Case #5: The patient is a 54-year-old man with hypertension using hydrochlorothiazide 25 mg/day and enalapril 10 mg/day. A computed tomography scan obtained to investigate abdominal pain identified a 1.7 cm nodule in the left adrenal gland with a benign appearance (low attenuation coefficient [8 HU], well-defined limits, rapid contrast washout). On physical examination, his blood pressure is 124/78 mmHg (right arm, lying down and sitting up).

**Table 1:** Indications for screening for primary aldosteronism according to the Endocrine Society guideline (9)

Sustained blood pressure above 150/100 mmHg on each of three measurements obtained on different days
Hypertension (blood pressure 140/90 mmHg) resistant to three conventional antihypertensive drugs (including a diuretic)
Controlled blood pressure (<140/90 mmHg) on four or more antihypertensive drugs
Hypertension and spontaneous or diuretic-induced hypokalemia
Hypertension and adrenal incidentaloma
Hypertension and sleep apnea
Hypertension and a family history of early onset hypertension or cerebrovascular accident at a young age (<40 years)
All hypertensive first-degree relatives of patients with primary hyperaldosteronism

The respondents were invited to analyze each case scenario and inform whether they would screen the patient for PHA. If the answer to PHA screening was negative, the respondents were asked to explain the reason for their decision. They were presented with the following options as reasons for not choosing to screen: "The antihypertensive medications in use can interfere with screening tests for primary hyperaldosteronism," "Screening is not recommended in this situation," "I do not have the necessary tests (aldosterone and plasma renin activity) at my workplace," and "Other reason."

The email with the link to the survey was sent weekly for 4 weeks, and after that, the survey was closed for responses. The responses were downloaded as an Excel file for analysis.

### Statistical analysis

The statistical analyses were performed using SPSS version 23.0 for MacOS (SPSS Inc., Chicago, IL, USA). In the descriptive analysis, categorical variables were expressed as frequency and percentage, while numerical variables were expressed as mean ± standard deviation. The distribution of the numerical variables was analyzed with the Kolmogorov-Smirnov test, which showed that all variables were normally distributed. Student's *t* test was performed to compare numerical variables between two groups, and analysis of variance (ANOVA) was used to compare numerical variables among three groups. In these cases, Tukey's *post hoc* test was used to identify which pairs of groups differed from each other. The chi-square test or Fisher's exact test was applied to compare categorical variables, as appropriate. Binary logistic regression was used to assess predictors to recommend PHA screening. A p value <0.05 was considered significant.

## RESULTS

In all, 126 physicians answered the online survey voluntarily. Data regarding their age and time since graduation and specialization are presented in Table 2. Among the medical specialties, 57.9% of the respondents were endocrinologists, 27.8% were cardiologists, and 14.3% were physicians from other specialties (this category included clinicians, nephrologists, and gastroenterologists). The mean age was higher among cardiologists than endocrinologists and physicians from other specialties (p < 0.001). The mean time since graduation and specialization was also longer among cardiologists compared with others (p < 0.001). The percentages of physicians practicing in the public or private sectors were comparable across medical specialties.

**Table 2:** Characteristics of the study participants

	Medical Specialties			
	Endocrinologists (n = 73)	Cardiologists (n = 35)	Others (n = 18)	P value*
Age (years)	43.1 ± 11.1†	54.0 ± 11.2† ‡	45.8 ± 15.2‡	<0.001
Time since graduation (years)	18.8 ± 11.0†	29.1 ± 10.7† ‡	20.5 ± 15.0‡	<0.001
Time since specialization (years)	14.71 ± 11.94†	25.11 ± 11.67†	17.39 ± 15.67	<0.001

The data were normally distributed and are presented as mean (± standard deviation) values.

* *P* value: analysis of variance (ANOVA) comparing all three groups. Tukey's *post hoc* test was used to identify which pairs of groups differed from each other.

† Cardiologists vs. endocrinologists;

‡ Cardiologists vs. physicians from other specialties ("others").

**Figure 1:**
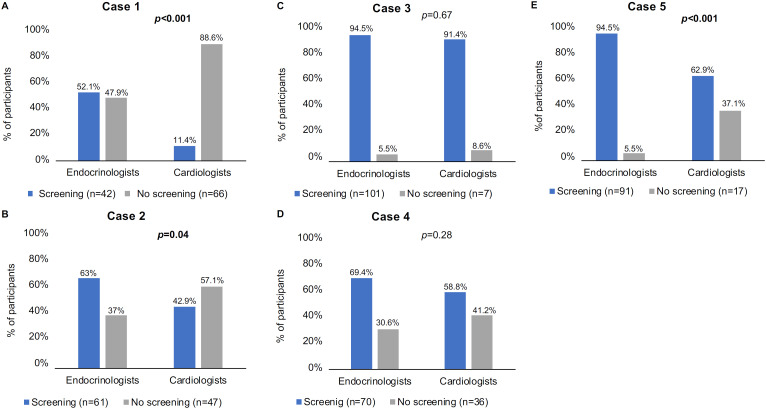
Percentages by medical specialties of respondents who chose vs. not chose screening for primary hyperaldosteronism in the five clinical cases (endocrinologists vs. cardiologists).

**Figure 2:**
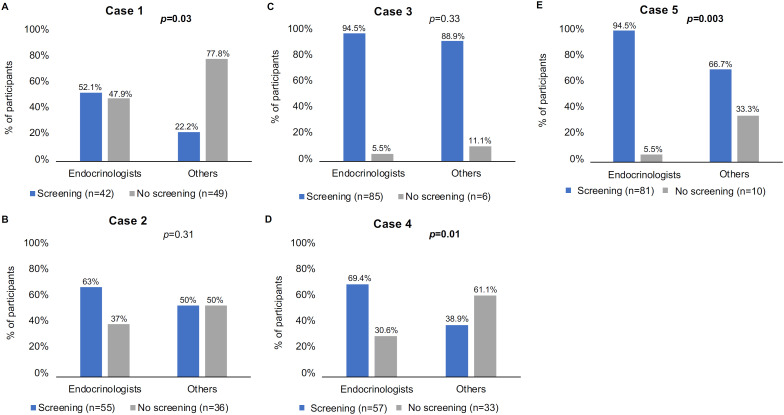
Percentages by medical specialties of respondents who chose vs. not chose screening for primary hyperaldosteronism in the five clinical cases (endocrinologists vs. physicians from other specialties ["others"]).

**Figure 3:**
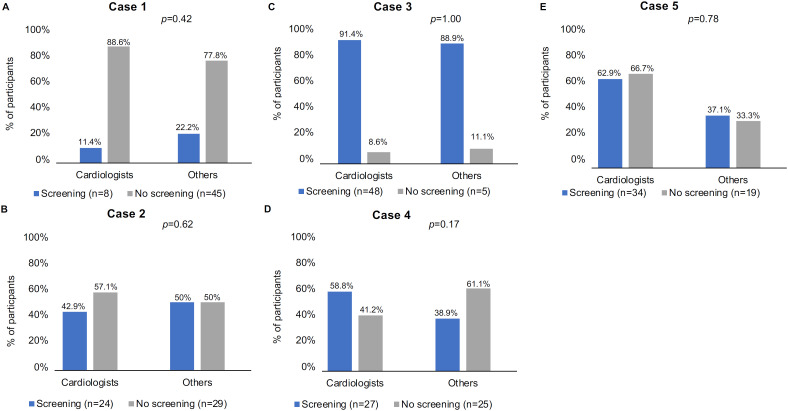
Percentages by medical specialties of respondents who chose vs. not chose screening for primary hyperaldosteronism in the five clinical cases (cardiologists vs. physicians from other specialties ["others"]).

Overall, 36.5%, 70%, 92.9%, 62.1%, and 81.7% of the respondents would recommend PHA screening for the patients in Cases #1, #2, #3, #4, and #5, respectively. The rates of screening recommendation in Cases #1, #2, and #5 were higher among endocrinologists than cardiologists (p < 0.001, p = 0.04, and p < 0.001, respectively) (Figure 1). Additionally, the rates of screening recommendation in Cases #1, #4, and #5 were higher among endocrinologists than specialists other than cardiologists (p = 0.03, p = 0.01, and p = 0.001, respectively) (Figure 2). Excluding endocrinologists, no significant differences in rates of screening recommendations were observed between cardiologists and physicians from other specialties across all cases (Figure 3).

Table 3 shows the reasons for not choosing PHA screening in each hypothetical situation according to specialty.

**Table 3:** Reasons for not choosing screening for primary hyperaldosteronism by medical specialty

		Number of participants who chose no screening	Tests not available (n)	Screening not indicated (n)	Interference from antihypertensive medications (n)	Others (n)
Case 1						
	Endocrinologists	35	2.4% (1)	75.7% (31)	17.1% (7)	4.8% (2)
	Cardiologists	31	-	68.6% (24)	28.6% (10)	2.8% (1)
	Others	14	12.5% (2)	68.7% (11)	12.5% (2)	6.3% (1)
Case 2						
	Endocrinologists	27	81.5% (22)	11.1% (3)	-	7.4% (2)
	Cardiologists	20	86.4% (19)	13.6% (3)	-	-
	Others	9	90% (9)	10% (1)	-	-
Case 3						
	Endocrinologists	4	25% (1)	-	75% (3)	-
	Cardiologists	3	-	-	100% (3)	-
	Others	2	-	50% (1)	-	50% (1)
Case 4						
	Endocrinologists	22	5% (1)	85% (17)	5% (1)	5% (1)
	Cardiologists	14	7.1% (1)	61.5% (8)	15.4% (2)	15.4% (2)
	Others	11	10% (1)	90% (9)	-	-
Case 5						
	Endocrinologists	4	25% (1)	25% (1)	50% (2)	-
	Cardiologists	13	15.4% (2)	53.8% (7)	30.8% (4)	-
	Others	6	-	100% (6)	-	-

Some respondents did not answer their reason for not choosing screening for primary hyperaldosteronism (Case #4), while others answered more than one reason for that (Cases #1 and #2).

Binary logistic regression analysis was performed to identify the independent variables associated with PHA screening, considering the outcome as the dependent variable. The variables included in the model were age, time since graduation, time since specialization, and medical specialty (endocrinology vs. cardiology). According to the model and considering all these four variables simultaneously, being an endocrinologist was an independent predictor of choosing PHA screening for the patients in Cases #1 and #5 (p = 0.001 and p = 0.002, respectively).

## DISCUSSION

It is well recognized that PHA remains an underdiagnosed condition. According to the 2016 Endocrine Society guideline (9), PHA is a "major public health issue requiring immediate recognition and coordinated action". Indeed, studies show that the percentage of patients who undergo PHA screening among those who are eligible for screening is below 3% in the US (4,5,8) and below 8% in Italy and Germany (6,7). Therefore, for the proper design of strategies to increase PHA awareness, diagnosis, and treatment in our country, it is essential to evaluate the screening practices and understand the reasons why screening is not conducted when recommended. To the best of our knowledge, this is the first study evaluating PHA screening practices in Brazil. The relevance of the study is emphasized by the overall low screening rates found among the specialties that most often treat patients with PHA (endocrinology and cardiology), even though all the clinical cases in the survey presented indications for PHA screening according to the 2016 Endocrine Society guideline (9).

Specifically, regarding screening rates and reasons for not screening, Case #3 had the highest screening rate (92.9%) among all specialists. This unsurprising finding was probably because this case presented a patient with a classic PHA phenotype, as described originally by Conn (11), *i.e.*, with resistant hypertension and hypokalemia. In this "classic" clinical case, the few respondents who chose not to screen for PHA, did so primarily out of concern that the antihypertensive drugs used by the patient could interfere with the screening tests. It is important to note that the patient in Case #3 was not using a mineralocorticoid antagonist, which is the main drug that interferes with the screening tests. Additionally, PHA screening tests do not require modification in antihypertensive medications (9) since (A) most interfering factors result in false-negative tests and, therefore, a positive test makes the diagnosis even more likely and (B) confirmatory tests can later rule out a false-positive result. While it is reassuring that most respondents would choose PHA screening for a typical patient like the one described in Case #3, it is important to note that hypokalemia is present in far less than half of the patients with PHA (12,13); therefore, cases with hypokalemia are only "the tip of the iceberg" when it comes to PHA. It is also not surprising that the frequency of PHA screening for Case #5 was higher among endocrinologists, since adrenal incidentaloma is a condition typically assessed by this medical specialty.

The percentages of respondents who would choose PHA screening for the patients in Cases #1, #2, and #4 were overall low (36.5%, 70%, and 62.1%, respectively). In Cases #1 and #4, the main reason for most respondents not choosing to screen was the lack of recognition of the clinical situation as an indication for screening. This represents a major challenge in PHA management, as these hypothetical patients probably represent most PHA cases. Considering that the screening rates were low despite the respondents being directed to consider the possibility of PHA in this research scenario (*i.e*., information bias), the screening rates are expected to be much lower in a real-life situation. This emphasizes the importance of raising awareness of PHA through scientific meetings and publications in scientific journals. Notably, a brief search of articles published in the official journal of the Brazilian Society of Endocrinology and Metabolism using the keywords "primary hyperaldosteronism", "primary aldosteronism", and "aldosteronoma" retrieved only 11 articles in the last 22 years, indicating an opportunity for action.

Another major barrier to PHA screening is the limited availability of tests within public health services. Indeed, this was the main reason given by respondents who chose no PHA screening for the patient in Case #2. An informal survey conducted among chiefs of endocrinology units in 11 public institutions in Rio de Janeiro revealed that none of the units have technical resources to measure aldosterone and renin in their local laboratories, and only three have agreements with private laboratories to carry out these measurements. This indicates another opportunity for potential strategies to encourage screening practices for patients with hypertension, considering that adequate PHA diagnosis and treatment mitigate cardiovascular risks and reduce health care services utilization (along with costs) and patients’ morbidity and mortality (1).

Cohen and cols. (8) also found a low PHA screening rate (1.6%) of US veterans with resistant hypertension. In their study, the factors associated with a greater likelihood of testing at the patient level were the presence of hypokalemia and higher blood pressure levels. At the provider level, a first visit with an endocrinologist or nephrologist, but not with a cardiologist, increased the likelihood of testing relative to a first visit with a primary care physician. This finding corroborates those of the present study, in which being an endocrinologist was an independent predictor of PHA screening when the screening rates were compared between specialties and on logistic regression analysis of independent variables associated with PHA screening. Recently, Hundemer and cols. (14) showed a PHA screening rate of only 3.9%, even when hypertension was associated with severe hypokalemia (potassium levels < 3.0 mEq/L). The authors hypothesized that, in addition to failure in recognizing indications to screen a patient for PHA, the low screening rates could also be due to a lack of recognition of the cardiovascular risk in excess to the risk from high blood pressure levels in patients with PHA and the complexity of the diagnostic algorithm (14). Considering these data, along with the findings of our study, it becomes imperative to understand the reasons preventing physicians from choosing PHA screening.

Long-standing undiagnosed PHA frequently leads to aldosterone-specific cardiovascular morbidity (myocardial infarction, stroke, coronary artery disease, and arrhythmias) and nephrotoxicity (15,16). An important meta-analysis of 31 studies including 3,838 patients with PHA and 9,284 patients with essential hypertension showed a significant increase in target-organ damage (left ventricular hypertrophy), metabolic syndrome, diabetes, and cardiovascular and cerebrovascular events in patients with PHA compared with those with essential hypertension, independent of blood pressure level (1).

Considering an estimated Brazilian population of 203 million people (17) and a hypertension rate of 32% (18), the estimated number of individuals with hypertension in the country is 65 million; of these, 3.25-6.5 million may have PHA (estimated rate 5%-10%) (18,19). These numbers should serve as a warning of the importance of detecting patients with potential PHA in order to minimize the onset of PHA complications and mitigate costs to the health system.

The 2016 Endocrine Society guideline on PHA recommends case detection of PHA in patients with sustained blood pressure >150/100 mmHg, hypertension (blood pressure >140/90 mmHg) resistant to three conventional antihypertensive drugs (including a diuretic), controlled blood pressure (<140/90 mmHg) on four or more antihypertensive drugs, hypertension and spontaneous or diuretic-induced hypokalemia, hypertension and adrenal incidentaloma, hypertension and sleep apnea, hypertension and family history of early-onset hypertension or cerebrovascular accident at a young age (<40 years), and all hypertensive first-degree relatives of patients with PHA (9). On the other hand, Japanese guidelines and some experts suggest that all patients with hypertension should be screened for PHA, regardless of pretest probability (10,20,21). Some current evidence supports the importance of early and systematic PHA screening in most, if not all, patients with hypertension to allow direct targeted pharmacotherapy for PHA to reverse excess cardiovascular risk (1). Additionally, clinicians should perform case detection testing for PHA at least once in all patients with hypertension (16).

Our study has some limitations. The first is its small number of participants and the fact that the sample was restricted to one Brazilian state. However, even though the number of participants was small, it exceeded the sample size calculated based on the number of specialists in the state. Second, considering the research scenario specifically aimed at PHA screening, the participants answered the survey already considering the possibility of this diagnosis, which could configure an information bias. Thus, the PHA screening rate in real-world practice would certainly be much lower. Finally, an important aspect is that we did not include members of the Nephrology Society of Rio de Janeiro (*Sociedade de Nefrologia do Rio de Janeiro*). Although their inclusion would have probably not resulted in higher screening rates, it would have been interesting to compare their responses with those from members of the other specialties included in the study.

In conclusion, endocrinologists were the specialists who most often chose PHA screening, although the screening rates were overall low among all specialists. These findings highlight a need for continuing medical education programs addressing PHA screening and making the diagnosis of PHA more present in the daily clinical practice of physicians treating patients with hypertension. This change would decrease the cardiovascular risk of patients with PHA, consequently reducing these patients’ morbidity and mortality and decreasing the costs to the health system.
